# P‐wave parameters and their association with thrombi and spontaneous echo contrast in the left atrial appendage

**DOI:** 10.1002/clc.23980

**Published:** 2023-02-17

**Authors:** Fabienne Kreimer, Julian Felix Backhaus, Christos Krogias, Andreas Pflaumbaum, Andreas Mügge, Michael Gotzmann

**Affiliations:** ^1^ Cardiology and Rhythmology, St Josef‐Hospital Ruhr University Bochum Bochum Germany; ^2^ Neurology, St Josef‐Hospital Ruhr University Bochum Bochum Germany

**Keywords:** atrial cardiomyopathy, atrial fibrillation, ischemic stroke, P‐wave parameters, spontaneous echo contrast, thrombus

## Abstract

**Background:**

The aim of this study was to examine the prevalence of abnormal P‐wave parameters in patients with thrombus and/or spontaneous echo contrast (SEC) in the left atrial appendage (LAA), and to identify P‐wave parameters particularly associated with thrombus and SEC formation.

**Hypothesis:**

We presume a significant relationship of P‐wave parameters with thrombi and SEC.

**Methods:**

All patients in whom a thrombus or SEC was detected in the LAA on transoesophageal echocardiography were included in this study. Patients at risk (CHA2DS2‐VASc Score ≥3) and routine transoesophageal echocardiography to exclude thrombi served as the control group. A detailed ECG analysis was performed.

**Results:**

Of a total of 4062 transoesophageal echocardiographies, thrombi and SEC were detected in 302 patients (7.4%). Of these patients, 27 (8.9%) presented with sinus rhythm. The control group included 79 patients. There was no difference in mean CHA2DS2‐VASc score in the two groups (*p* = .182). A high prevalence of abnormal P‐wave parameters was detected in patients with thrombus/SEC. Indicators for the presence of thrombi or SEC in the LAA were P‐wave duration >118 ms (Odds ratio (OR) 3.418, Confidence interval (CI) 1.522–7.674, *p* < .001), P‐wave dispersion >40 ms (OR 2.521, CI 1.390–4.571, *p* < .001) and advanced interatrial block (OR 1.431, CI 1.033–1.984, *p* = .005).

**Conclusion:**

Our study revealed that several P‐wave parameters are associated with thrombi and SEC in the LAA. The results may help identify patients who are at particularly high risk for thromboembolic events (e.g., in patients with embolic stroke of undetermined source).

## Graphical abstract

Several P‐wave parameters are associated with atrial fibrillation and with ischemic stroke. However, it is uncertain whether P‐wave parameters are associated with thrombus or spontaneous echo contrast in the left atrial appendage. Our study demonstrated a significant association of P‐wave duration, P‐wave dispersion, and advanced interatrial block with thrombus or spontaneous echo contrast in the left atrial appendage. IAB, interatrial block; PTFV1, P‐wave terminal force in V1.

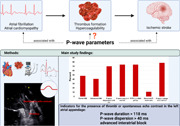

**Abbreviations**
AF: atrial fibrillation
AUC: area under the curve
CI: confidence interval
ECG: electrocardiography
IAB: interatrial block
LAA: left atrial appendage
OR: odds ratio
PTFV1: P‐wave terminal force in V1
SEC: spontaneous echo contrast
TIA: transient ischemic attack

## 1 INTRODUCTION

Alterations in P‐wave parameters can reflect structural, functional, as well as electrical remodeling of the atria.^1^ In the past, associations of abnormal P‐wave parameters with clinical outcomes, particularly atrial fibrillation (AF) and ischemic stroke, have been reported.^1^ In addition, P‐wave parameters are useful in the diagnosis of atrial cardiomyopathy which is defined as as “any complex of structural, architectural, contractile or electrophysiological changes affecting the atria with the potential to produce clinically‐relevant manifestations.”^1^, ^2^ Patients with embolic stroke of undetermined source (ESUS) and patients with AF are particularly interesting because atrial cardiomyopathy is often presumed to be the underlying disease.^1^ Thromboembolic events resulting from thrombus formation, predominantly in the left atrial appendage (LAA), may occur as a result of both AF and atrial cardiomyopathy, independent of AF. Thus, the development of thrombus and spontaneous echo contrast (SEC) in the LAA may be the causal link between AF and/or atrial cardiomyopathy and ischemic stroke.

Thrombi and SEC in the LAA are relatively common findings on transesophageal echocardiography, particularly in patients with AF.^3^ Besides the increased risk of thromboembolic events, both phenomena are associated with higher morbidity and mortality.^4^, ^5^, ^6^, ^7^, ^8^, ^9^ Furthermore, the occurrence of thrombus or SEC is considered as an additional diagnostic criterion for the presence of atrial cardiomyopathy, characterized by functional and structural atrial dysfunction.^10^, ^11^ However, there is limited evidence on the association of electrical dysfunction of the atrium and the occurrence of thrombi and SEC on transoesophageal echocardiography.

Consequently, because thrombus formation and SEC could be the link between AF, atrial cardiomyopathy, and ischemic stroke, the question arises whether there are P‐wave indices associated with thrombus and SEC. Therefore, the aim of this study was to examine the prevalence of abnormal P‐wave parameters in patients with thrombus/SEC, and to identify P‐wave parameters particularly associated with thrombus and SEC formation.

## 2 METHODS

This study examined the P‐wave indices of patients with evidence of thrombus/SEC in the LAA compared with the P‐wave indices of patients with comparable stroke risk and exclusion of thrombus or SEC. For the analysis of P‐wave indices, it was necessary that the studied patients were in sinus rhythm.

Medical history, laboratory examinations, echocardiography results, oral anticoagulation, and ECG analysis were recorded for all patients before transoesophageal echocardiography. This study was a retrospective analysis of prospectively obtained data. All examinations were performed at the St Josef Hospital—Hospital of the Ruhr University Bochum. Patients gave informed consent. The study was approved by the local ethics committee of the Ruhr University Bochum (Number 22‐7565).

### 2.1 Inclusion and exclusion criteria

The study population was composed of a thrombus/SEC group and a control group. For the thrombus/SEC group, we examined retrospectively all transoesophageal echocardiographies performed between 2015 and 2020.^12^ All patients with sinus rhythm, in whom a thrombus or a SEC in the LAA were detected were included in the study. The indications for transoesophageal echocardiography were planned pulmonary vein isolation, in cases of ESUS, and for evaluation of valvular heart diseases, or unclear infections. Patients with other rhythm than sinus rhythm (e.g., AF, atrial flutter), thrombi in other cardiac cavities, cardiac tumors and previous interventional or surgical occlusion of the LAA were excluded.

To assess the significance of P‐wave indices for association with thrombus or SEC, we identified a control group with the following characteristics: (1) exclusion of thrombus/SEC in the LAA on transoesophageal echocardiography, (2) presence of sinus rhythm at the time of transoesophageal echocardiography so that P‐wave indices could be compared, (3) a comparable increased risk of stroke, as measured by an elevated CHA_2_DS_2_‐VASc score (CHA_2_DS_2_‐VASc score ≥3), (4) exclusion of severe concomitant valvular heart disease, (5) exclusion of patients with AF, atrial flutter, thrombi in other cardiac cavities, cardiac tumors and previous interventional or surgical occlusion of the LAA.

### 2.2 Echocardiographic analysis

Transthoracic and transoesophageal echocardiography were performed with a digital ultrasound scanner (Vivid E9; General Electric). Transoesophageal echocardiography was conducted by experienced physicians according to current recommendations.^13^ Thrombus in the LAA was defined as a solid echo dense structure that was detectable in multiple image planes in the LAA.^14^ SEC in the LAA was diagnosed when swirling dynamic echoes were detected without evidence of thrombus^14^ (Supporting Information: Figure 1).

### 2.3 Electrocardiographic analysis

All patients received a 12‐lead ECG performed within 24 h before transoesophageal echocardiography. The standard 12‐lead surface ECG was recorded at a rate of 50 mm/s and a voltage of 10 mm/mV. All evaluations were conducted by a single observer who was blinded to the patients' group. The focus of the ECG analysis was on the examination of the P‐wave.

The duration of the P‐wave was determined in all 12 leads. The resulting arithmetic average represented the mean P‐wave duration. P‐wave dispersion was calculated by subtracting the shortest P‐wave duration from the longest P‐wave duration.^1^ A P‐wave dispersion of 40 ms is associated with worse clinical outcome, such as the occurrence of AF, and was therefore considered as abnormal.^15^ A prolonged P‐wave duration ≥120 ms is pathological and represents a partial IAB which indicates delayed excitation conduction between the right and left atrium via the Bachmann bundle.^1^ If the Bachmann bundle is completely blocked, there is an advanced IAB that consists not only of a prolonged P‐wave duration ≥120 ms, but also of an additional biphasic morphology in the inferior leads due to retrograde excitation of the left atrium.^1^ A biphasic P‐wave is often found in lead V1. The terminal negative portion represents excitation in the left atrium. Left atrial enlargement may be reflected by an abnormal P‐wave terminal force in lead V1 (PTFV1). It is calculated by multiplying the terminal portion of the P‐wave in V1 by the duration of this portion. A PTFV1 ≤ −4000 µV*ms is considered pathological.^1^ The patients' ECG were examined for a P‐wave voltage ≤0.1 mV in lead I, which is defined as abnormal.^1^ The P‐wave area was measured by multiplying half of the P‐wave duration in lead II by the P‐wave voltage in this lead, and is considered pathological at a value of at least 4 ms*mV.^1^ The P‐wave axis was determined. The normal P‐wave axis is a value between 0° and +75°. Deviations from this axis were defined as abnormal P‐axis^1^ (Supporting Information: Figure 2).

## 3 STATISTICS

The statistical software SPSS 26 was used for statistical analysis. Numerical values are expressed as mean ± SD. Continuous variables were compared between groups using an unpaired *t‐*test (for normally distributed variables) or Mann–Whitney *U*‐test (for non‐normally distributed variables). *χ* ^2^ analysis was used to compare categoric variables. Receiver operating characteristic curves were generated to define cutoff values for the mean P‐wave duration, P‐wave dispersion and PTFV1. The odds ratio was measured for risk calculation. A *p* < .05 was considered significant. All probability values reported are 2‐sided.

## 4 RESULTS

Between 2015 and 2020, 4062 transoesophageal echocardiographies were performed at the university hospital St Josef‐Hospital Bochum. Thrombi in the LAA were detected in 51 patients (1.2%) and SEC in the LAA in 251 patients (6.2%). Of these patients, 27 (8.9%) had sinus rhythm at the time of transoesophageal echocardiography. These patients formed the thrombus/SEC group (*n* = 27). In this group, five patients had thrombus (each with SEC), two patients had sludge (with SEC), and the remaining patients had SEC. The indications for transoesophageal echocardiography in the thrombus/SEC group were planned pulmonary vein isolation (*n* = 11), in cases of ESUS (*n* = 9), for evaluation of valvular heart diseases (*n* = 4), and unclear infections (*n* = 3).

The control group consisted of 79 patients without thrombus/SEC and in sinus rhythm at the time of transoesophageal echocardiography with a CHA_2_DS_2_‐VASc score ≥3. The indication for transoesophageal echocardiography in all patients (*n* = 79) of the control group who met the inclusion criteria was planned pulmonary vein isolation. Accordingly, our final study population consisted of 106 patients. The mean age of the study population (59% women, 41% men) was 70 ± 8.5 years (minimum 47 years, maximum 86 years). The mean left ventricular ejection fraction was 55 ± 10.8% and the CHA_2_DS_2_‐VASc score was 4 ± 1.3 points.

### 4.1 Patients' clinical characteristics

Patients with thrombus/SEC were significantly more likely to have diabetes mellitus and to have a history of stroke/transient ischemic attack (TIA) than the control group. While 20 patients (74%) in the thrombus/SEC group had a history of AF, all patients (100%) in the control group presented with a history of AF (*p* < .001). Accordingly, it is not surprising that the control group was significantly more likely to take oral anticoagulation. Otherwise, there were no significant differences between the patient groups regarding laboratory parameters (e.g., creatinine, hemoglobin), left atrial diameter, left ventricular ejection fraction or CHA_2_DS_2_‐VASc score (Table 1).

**Table 1:** Characteristics of the study population (*n* = 106).

	Thrombus/SEC group (*n* = 27)	Control group (*n* = 79)	*p* Value
Age (years)	70 ± 10	70 ± 8	.726
CHA_2_DS_2_‐VASc‐Score, pts	4.4 ± 1.7	3.9 ± 1.1	.182
Congestive heart failure, *n* (%)	9 (33)	18 (23)	.327
Hypertension, *n* (%)	27 (100)	73 (92)	.459
Diabetes mellitus, *n* (%)	11 (41)	13 (16)	.013
Stroke/TIA, *n* (%)	11 (41)	14 (18)	.020
Vascular disease, *n* (%)	14 (52)	37 (47)	.773
Women (♀), *n* (%)	14 (52)	49 (62)	.266
Atrial fibrillation, *n* (%)	20 (74)	79 (100)	<.001
*COPD, n (%)*	2 (7)	10 (13)	.427
Echocardiography			
Left ventricular ejection fraction (%)	52 ± 13	56 ± 10	.100
Left atrial diameter (mm)	42 ± 7	41 ± 5	.749
Mitral valve regurgitation, *n* (%)	23 (85)	65 (82)	.507
Labor			
Creatinine (mg/dl)	1.1 ± 0.3	1.0 ± 0.2	.420
Hemoglobin (g/dl)	12.9 ± 2.2	13.4 ± 1.5	.190
Oral anticoagulation, *n* (%)	13 (48)	78 (99)	<.001
ECG indices			
Heart rate (beats/min)	70 ± 13	66 ± 11	.118
P‐wave duration mean (ms)	127 ± 18	113 ± 19	.001
P‐wave axis (°)	56 ± 20	54 ± 20	.541
P‐wave dispersion (ms)	43 ± 12	27 ± 14	<.001
P‐wave amplitude in I < 0.1 mV, *n* (%)	21 (78)	52 (66)	.251
P‐wave area (mV*ms)	7.3 ± 3.8	6.6 ± 2.8	.333
P‐wave terminal force in V1 (µV*ms)	−7593 ± 4958	−4810 ± 3048	.001
Partial interatrial block, *n* (%)	13 (48)	30 (38)	.353
Advanced interatrial block, *n* (%)	11 (41)	12 (15)	.005
PR interval (ms)	195 ± 45	180 ± 35	.076
QRS duration (ms)	105 ± 22	100 ± 19	.251
QT interval (ms)	428 ± 46	422 ± 34	.443
QRS axis (°)	11 ± 44	17 ± 38	.493
T‐wave axis (°)	54 ± 60	49 ± 35	.664

Abbreviations: COPD, chronic obstructive pulmonary disease; SEC, spontaneous echo contrast.

### 4.2 Prevalence of abnormal P‐wave indices in the study population

**Figure 1:**
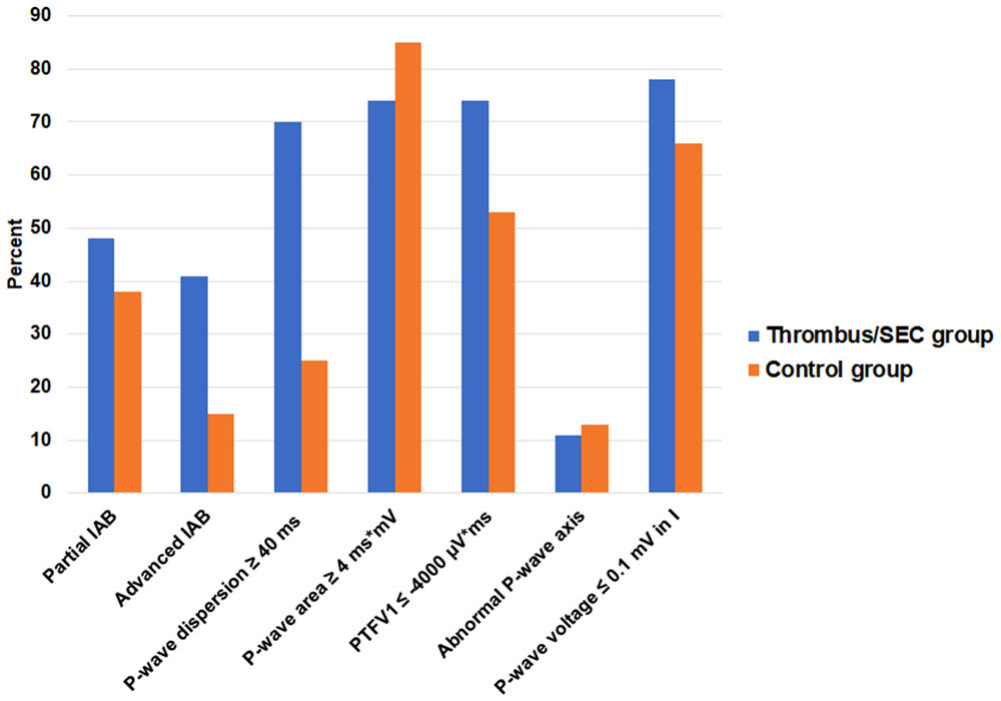
Prevalences of abnormal P‐wave indices in the thrombus/spontaneous echo contrast group and in the control group. IAB, interatrial block; PTFV1, P‐wave terminal force in V1.

Both groups were examined for the presence of abnormal P‐wave indices. In the thrombus/SEC group, 13 patients (48%) had partial, and 11 patients (41%) had advanced IAB. A P‐wave dispersion of at least 40 ms was present in 19 patients (70%), and a PTFV1 ≤ −4000 µV*ms in 20 patients (74%). Also 20 patients (74%) had a P‐wave area of at least 4 ms*mV, and a P‐wave voltage ≤0.1 mV in lead I was detected in 21 patients (78%), respectively. In addition, 3 patients (11%) exhibited a pathological P‐wave axis (Table 2, Figure 1). In the thrombus/SEC group, all patients had at least one combination of two abnormal P‐wave indices.

**Table 2:** Prevalence of abnormal P‐wave parameters in the thrombus/SEC group (*n* = 27) and in the control group (*n* = 79).

	Thrombus/SEC group (*n* = 27)	Control group (*n* = 79)	*p* Value
Partial interatrial block, *n* (%)	13 (48)	30 (38)	.353
Advanced interatrial block, *n* (%)	11(41)	12 (15)	.005
P‐wave dispersion ≥40 ms, *n* (%)	19 (70)	20 (25)	<.001
P‐wave terminal force in V1 ≤ −4000 µV*ms, *n* (%)	20 (74)	42 (53)	.091
P‐wave area ≥4 ms*mV, *n* (%)	20 (74)	67 (85)	.209
Abnormal P‐wave axis (<0°, >75°), *n* (%)	3 (11)	10 (13)	.831
P‐wave voltage ≤0.1 mV in I, *n* (%)	21 (78)	52 (66)	.247

Abbreviation: SEC, spontaneous echo contrast.

In the control group, 30 patients (38%) exhibited partial, and 12 patients (15%) advanced IAB. 20 patients (25%) had a P‐wave dispersion of at least 40 ms, and 42 patients (53%) had a PTFV1 ≤ −4000 µV*ms. In 67 patients (85%), there was a P‐wave area of at least 4 ms*mV, and in 52 patients (66%) a P‐wave voltage ≤0.1 mV in lead I was found. Furthermore, 10 patients (13%) displayed a pathological P‐wave axis (Table 2, Figure 1). In the control group, there was only one patient who had no abnormal P‐wave indices.

### 4.3 Comparison of electrocardiographic characteristics of the thrombus/SEC group and the control group

Several P‐wave indices showed significant differences between the thrombus/SEC group and the control group. Patients with thrombus/SEC were significantly more likely to present with a greater mean P‐wave duration, a greater P‐wave dispersion, and a more negative PTFV1 than the control group. Furthermore, these patients had also more often an advanced IAB. All other ECG parameters, in particular those related to ventricular excitation conduction and repolarization, demonstrated no significant differences (Table 1).

### 4.4 Indicators for the presence of thrombus/spontaneous echo contrast

**Figure 2:**
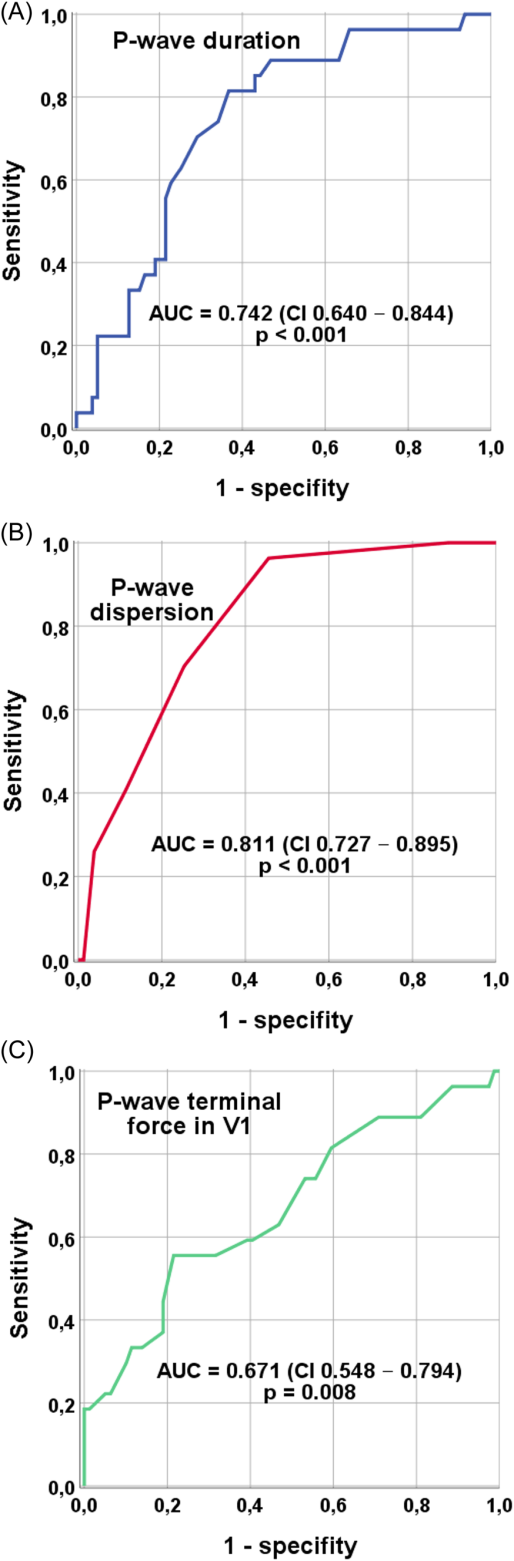
Receiver operating characteristics analysis for indication of thrombus/spontaneous echo contrast; (A) cutoff value P‐wave duration >118 ms (sensitivity 82%, specificity 63%); (B) cutoff value P‐wave dispersion >40 ms (sensitivity 70%, specificity 75%); (C) cutoff value PTFV1 <−4000 µV*ms (AUC 0.671, sensitivity 63%, specificity 63%). AUC, area under the curve; PTFV1, P‐wave terminal force in V1.

Using receiver operating characteristic analysis, optimal cutoff values for separating the study population were P‐wave duration >118 ms (area under the curve (AUC) 0.742, sensitivity 82%, specificity 63%, *p* < .001), P‐wave dispersion > 40 ms (AUC 0.811, sensitivity 70%, specificity 75%, *p* < .001) and PTFV1 < −4000 µV*ms (AUC 0.671, sensitivity 63%, specificity 63%, *p* = .008) (Figure 2).

The risk calculation identified P‐wave duration >118 ms (OR 3.418, CI 1.522–7.674, *p* < .001), P‐wave dispersion >40 ms (OR 2.521, CI 1.390–4.571, *p* < .001) and advanced IAB (OR 1.431, CI 1.033–1.984, *p* = .005) as indicators for thrombus/SEC in the transoesophageal echocardiography (Table 3). PTFV1 < −4000 µV*ms did not demonstrate a significant risk increase (OR 1.807, CI 0.916–3.564, *p* = .057).

### 4.5 Subgroup analysis of patients with oral anticoagulation

For a subgroup analysis, we included all patients with oral anticoagulation (*n* = 91). The thrombus/SEC group (*n* = 13) exhibited a significant lower left ventricular ejection fraction than the control group (*n* = 78), and higher creatinine and lower hemoglobin levels (Supporting Information: Table 1). However, significant differences in the P‐wave indices were also observed in the subgroup analysis. Patients with a thrombus/SEC had a significant longer mean P‐wave duration than the control group, a greater P‐wave dispersion, and a more negative PTFV1. There was no significant difference in the presence of a partial or advanced IAB between both groups (Supporting Information: Table 2).

## 5 DISCUSSION

In the present study, ECG parameters and their association with thrombi/SEC in the LAA were investigated. The main findings of our study are that in a group of high‐risk patients for stroke, some P‐wave indices are associated with the presence of thrombus/SEC. The risk for the occurrence of a thrombus/SEC increased significantly in the presence of a P‐wave duration >118 ms, a P‐wave dispersion >40 ms, and an advanced IAB (Table 3). Even in the subgroup analysis of patients with oral anticoagulation, significant differences in P‐wave indices were observed between the thrombus/SEC group and the control group (Supporting Information: Table 2).

In contrast, there were no significant differences regarding ECG parameters representing ventricular excitation propagation and regression (Table 1).

### 5.1 Diabetes mellitus and the presence of thrombus/spontaneous echo contrast

In the past, an association of diabetes mellitus and the occurrence of thrombus in the left atrium has been described by several studies.^16^, ^17^, ^18^, ^19^, ^20^, ^21^ In our study, the group with thrombi or SEC presented significantly more patients with diabetes mellitus, despite an overall similar CHA_2_DS_2_‐VASc score. This observation may support previous studies (Table 1). Hyperinsulinemia and hyperglycemia, particularly coexisting, have been demonstrated to cause a prothrombotic state. In addition, proinflammatory and proatherogenic processes induced by hyperglycaemia and hyperinsulinemia play an important role in thrombogenesis.^22^, ^23^ Furthermore, diabetes mellitus, accompanied by metabolic changes, insulin resistance, and hyperglycaemia, is associated with increased platelet activation and decreased response to antiplatelet agents.^23^

### 5.2 QRS width and the presence of left atrial appendage thrombus

Recently, Wegner et al.^24^ were able to demonstrate an association of QRS width and the presence of LAA thrombus and reduced LAA flow in 512 patients receiving transoesophageal echocardiography before cardioversion. However, in this study, left ventricular ejection fraction differed between the group with normal LAA flow and LAA thrombus (55% vs. 35%). Reduced ejection fraction is often associated with ventricular excitation propagation abnormalities, which may be reflected in widened QRS duration.^25^ In our study population, there was no significant difference regarding left ventricular ejection fraction (Table 1). A possible explanation might be the heterogeneity of the two study cohorts.

### 5.3 Prevalences of abnormal P‐wave indices in different populations

The P‐wave reflects the electrical excitation propagation of first the right and then the left atrium. For this reason, the P‐wave is particularly interesting regarding the evaluation of left atrial enlargement as well as intra‐ or interatrial conduction delays.

In the past, an association of abnormal P‐wave parameters with clinical outcomes (e.g., AF, thromboembolic events) has been described.^1^, ^26^, ^27^, ^28^, ^29^ For this reason, it is not surprising that abnormal P‐wave parameters are common in our study cohort, consisting of a very large proportion of patients with a history of AF (Table 1). Interestingly, however, for many pathological P‐wave indices, a higher prevalence was seen in patients with thrombus/SEC (Table 2).

Particularly in comparison to the general population, these prevalences are remarkable: epidemiologic findings indicate a prevalence of advanced IAB in the general population at 0.1%–0.5%, with an incidence of 2.27 per 1000 person‐years.^27^, ^30^ The prevalence of partial IAB is most likely even higher. In the large primary care population of the Copenhagen ECG Study, a P‐wave ≥120 ms was found in 20% of subjects.^30^, ^31^ In a representative Finnish population sample the prevalences of abnormal PTFV1, partial, and advanced IAB were 4.9%, 11.8%, and 1.9%.^32^ A Japanese study also demonstrated a prevalence for PTFV1 of 4.9%.^33^ In another Finnish cohort, incidence rates for prolonged P‐wave duration, abnormal PTFV1, left P‐wave axis deviation, and right P‐wave axis deviation were found to be 16.0%, 7.4%, 3.4%, and 2.2%, respectively.^34^ In the large Atherosclerosis Risk in Communities (ARIC) study, the prevalence of abnormal P‐wave axis was comparable at 8.3%.^35^

**Table 3:** P‐wave indices with significant association with thrombus or spontaneous echo contrast.

	Odds ratio	Confidence interval	*p* Value
P‐wave duration >118 ms	3.418	1.522–7.674	<.001
P‐wave dispersion >40 ms	2.521	1.390–4.571	<.001
Advanced interatrial block	1.431	1.033–1.984	.005

### 5.4 P‐wave indices and the risk of thrombus/spontaneous echo contrast

In this study cohort, P‐wave parameters could be derived from ECG analysis as indicators for the presence of thrombus/SEC. These findings suggest that abnormal ECG parameters as an expression of electrical dysfunction may be accompanied by concomitant mechanical dysfunction. However, it is not possible to conclude how the respective contribution of electrical and mechanical dysfunction to the thrombus formation is.

Nevertheless, our findings address a gap in the causal chain between AF and/or atrial cardiomyopathy, thrombus development, and ischemic stroke, because only an association of P‐wave indices and AF and/or atrial cardiomyopathy and ischemic stroke has been demonstrated so far. More interestingly, several P‐wave indices may indicate an increased risk of thrombus formation and thus may be helpful in risk stratification of patients with ESUS, and/or atrial cardiomyopathy.

Notably, in our study, the left atrial diameter as measured in the transthoracic echocardiography was not significantly different between the thrombus/SEC group and the control group (Table 1). On the one hand, this observation could indicate that a structural change of the left atrium (in the sense of enlargement) has no hemodynamic effects and thus does not lead to thrombus formation. On the other hand, it is striking that in both patient groups the left atrial diameters are enlarged, and thus it could be that both groups are similarly limited in left atrial hemodynamic. Therefore, the results of the ECG analysis are particularly interesting, as they emphasize the importance of the electrical excitation conduction in the atria for the development of a thrombus. In our study, a P‐wave duration >118 ms, a P‐wave dispersion >40 ms, and an advanced IAB were independent risk factors for the presence of thrombus/SEC in the transoesophageal echocardiography. However, there was an association between the PTFV1 and the presence of thrombus/SEC, but the risk calculation did not reveal a significantly increased risk. Okin et al.^36^ demonstrated that an abnormal PTFV1 correlated strongly with incident stroke in 7778 patients with hypertension. The authors suggested that an underlying subclinical atrial cardiomyopathy in the absence of AF might cause thrombus formation in the left atrium.^36^ Instead, our findings may imply a smooth transition because this parameter was also pathological in both groups. Furthermore, this observation reinforces the hypothesis that electrical remodeling of the atrium has a greater impact on thrombus formation than structural remodeling, because PTFV1 is considered as an important parameter for atrial enlargement.^1^

### 5.5 P‐wave duration

Normally, the P‐wave duration is less than 120 ms. In our study, the cutoff value with the highest sensitivity and specificity to discriminate between thrombus/SEC group and control group was 118 ms, which is close to the definition of a partial IAB (≥120 ms)^1^ (Table 3). Prolonged P‐wave duration indicates a delay in inter‐ and/or intra‐atrial excitation propagation.

Recently, Darweesh et al.^37^ demonstrated a negative correlation between P‐wave duration and global peak atrial longitudinal strain representing left atrial dysfunction. Fink et al.^38^ investigated 31 patients with wide‐area LAA isolation and subsequent LAA ligation. Interestingly, LAA thrombus was found in in 11 patients (35.5%) after wide‐area LAA isolation, but in no patient after LAA ligation.^38^ Moreover, the combination of wide‐area LAA isolation and LAA ligation reduced the P‐wave duration significantly.^38^ The study by Kawamura et al.^39^ supports these observations. Fifteen Patients with LAA ligation were analyzed for changes in P‐wave parameters after the procedure. P‐wave duration also decreased significantly in these patients.^39^

Huang et al.^40^ examining 128 patients with AF at risk for LAA thrombus, exhibited that the amplified P‐wave duration predicts left atrial thrombogenesis. They found an optimal threshold of 165 ms for amplified P‐wave duration with an AUC of 0.90, sensitivity of 88.5%, and specificity of 75.5%, respectively.^40^ Müller‐Edenborn et al.^41^ conducted a case‐control study dealing with amplified P‐wave duration for atrial cardiomyopathy and thromboembolism risk stratification. Stages of atrial cardiomyopathy were defined using amplified P‐wave duration. They demonstrated an OR for LAA thrombus of 24.6 (*p* < .001) per atrial cardiomyopathy stage.^41^

Prolonged P‐wave duration often indicates left atrial electrical dysfunction (the so‐called “P‐sinistroatriale”). The described studies as well as ours could show that mechanical dysfunction may also accompany it, increasing the risk for thromboembolism.

### 5.6 P‐wave dispersion

In the past, studies demonstrated an association of abnormal P‐wave dispersion with incident AF and AF recurrence after cardioversion.^42^, ^43^ More recently, the only independent predictor of AF detection was P‐wave dispersion of 40 ms in a study cohort of patients with cryptogenic stroke which were monitored by implantable loop recorders.^15^ In our study, we were able to demonstrate an association between an abnormal P‐wave dispersion ≥40 ms and the presence of thrombus/SEC.

In 2004, Dogan et al.^44^ investigated P‐wave dispersion in 64 patients undergoing cardioversion. Increased LA size and P‐wave dispersion were independent predictors of recurrent AF, while the presence of SEC was only in univariate analysis significant.^44^ In the same year, Güler et al.^45^ analyzed 44 patients with mitral stenosis. P‐wave dispersion and left atrial diameter turned out as strong predictors of LAA mechanical dysfunction reflected by low LAA peak emptying velocity.^45^

The previous described studies of Fink et al.^38^ and Kawamura et al.^39^ demonstrated that P‐wave dispersion decreased significantly after LAA ligation.

### 5.7 Advanced interatrial block

Advanced IAB is present if the P‐wave exhibits a duration ≥120 ms and simultaneously a biphasic morphology in the inferior leads.^1^ This occurs during complete block of interatrial excitation propagation via the Bachmann bundle.^1^ The presence of an advanced IAB is associated with worse clinical outcomes, for example, incident AF or all‐cause mortality after ischemic stroke.^27^, ^46^

In 2001, Goyal et al.^47^ identified IAB as a marker of left atrial electromechanical dysfunction. They analyzed 40 patients (24 patients with IAB, 16 patients without IAB) and demonstrated that the contractility of patients with IAB was significantly lower, presented as longer A‐wave acceleration times, and smaller left atrial stroke volumes, ejection fraction and kinetic energy.^47^

Ariyarajah et al.^48^ examined 66 patients with embolic stroke and sinus rhythm at ECG. Of those, 40 patients (61%) presented with IAB.^48^ Left atrial thrombi and/or SEC were found in 6 patients with IAB (15%), but in no patient without IAB.^48^ In our study, advanced IAB was an independent risk factor for the presence of thrombus or SEC on transoesophageal echocardiography (Table 3). However, it remains unclear why interatrial excitation delay is associated with mechanical dysfunction and thrombus formation in the left atrium.

## 6 LIMITATIONS

The main limitation of the present study is its retrospective and monocentric character. Therefore, it was not possible to evaluate the patients' atrial hemodynamic or laboratory parameters, which may also have influenced the outcome. Furthermore, the prevalence of abnormal P‐wave indices in our study cohort is high compared with the general population. Moreover, the thrombus/SEC group and the control group are heterogenous regarding the intake of oral anticoagulants. However, for this reason, we performed a subgroup analysis that included only patients with oral anticoagulation. Another limitation is the relatively small number of included patients.

## 7 CONCLUSION

Our findings address a previously existing gap between the known association of abnormal P‐wave indices with AF, atrial cardiomyopathy, and ischemic stroke. P‐wave parameters, which have the advantage of objectivity and broad, routine clinical application, may be suitable to predict the presence of thrombi and SEC on transoesophageal echocardiography. We were able to demonstrate a very high prevalence of abnormal P‐wave parameters in patients with thrombus/SEC. Even compared with high‐risk patients, the prevalence was higher for multiple parameters.

At the same time, P‐wave indices are markers of atrial cardiomyopathy. P‐wave indices could therefore help to identify patients at risk who suffer from atrial cardiomyopathy and are therefore particularly at risk for thrombus formation in the LAA and consequent thromboembolic events. In addition, the findings could allow risk stratification in patients with AF to differentiate which patients may be at particular risk for thrombus development and cardioembolic stroke.

Hence, the identified parameters could be of particular clinical importance for risk stratification of patients with AF, atrial cardiomyopathy, and ESUS. In general, patients with AF receive outpatient cardiological care, and patients with ESUS and atrial cardiomyopathy also receive cardiological evaluation. Herein, more attention should be paid to P‐wave parameters in routine ECG and changes in these parameters should also be monitored. Patients without previously diagnosed AF with atrial cardiomyopathy or ESUS who have risk factors according to the CHA_2_DS_2_‐VASc score in addition to abnormal P‐wave parameters could potentially benefit from therapy with oral anticoagulants. However, this has not yet been demonstrated in any large, randomized trial. In contrast, patients with known AF who already receive anticoagulation because of an increased CHA_2_DS_2_‐VASc score but who exhibit abnormal P‐wave parameters might benefit from closer echocardiographic monitoring and, if a thrombus is detected, from modification or escalation of therapy. This is particularly important in patients who present with a progressively lower left ventricular ejection fraction and thus are at greatly increased risk for cardiac thrombus formation, atrial as well as ventricular.

## Supporting information

Supporting information.

Supporting information.

Supporting information.

## Data Availability

Data available on request due to privacy/ethical restrictions.
