# Supplementary figures and images for: P‐wave parameters and their association with thrombi and spontaneous echo contrast in the left atrial appendage
Source: Clin Cardiol. 2023 Feb 17;46(4):397–406. doi: 10.1002/clc.23980 (PMC10106666; doi:10.1002/clc.23980)

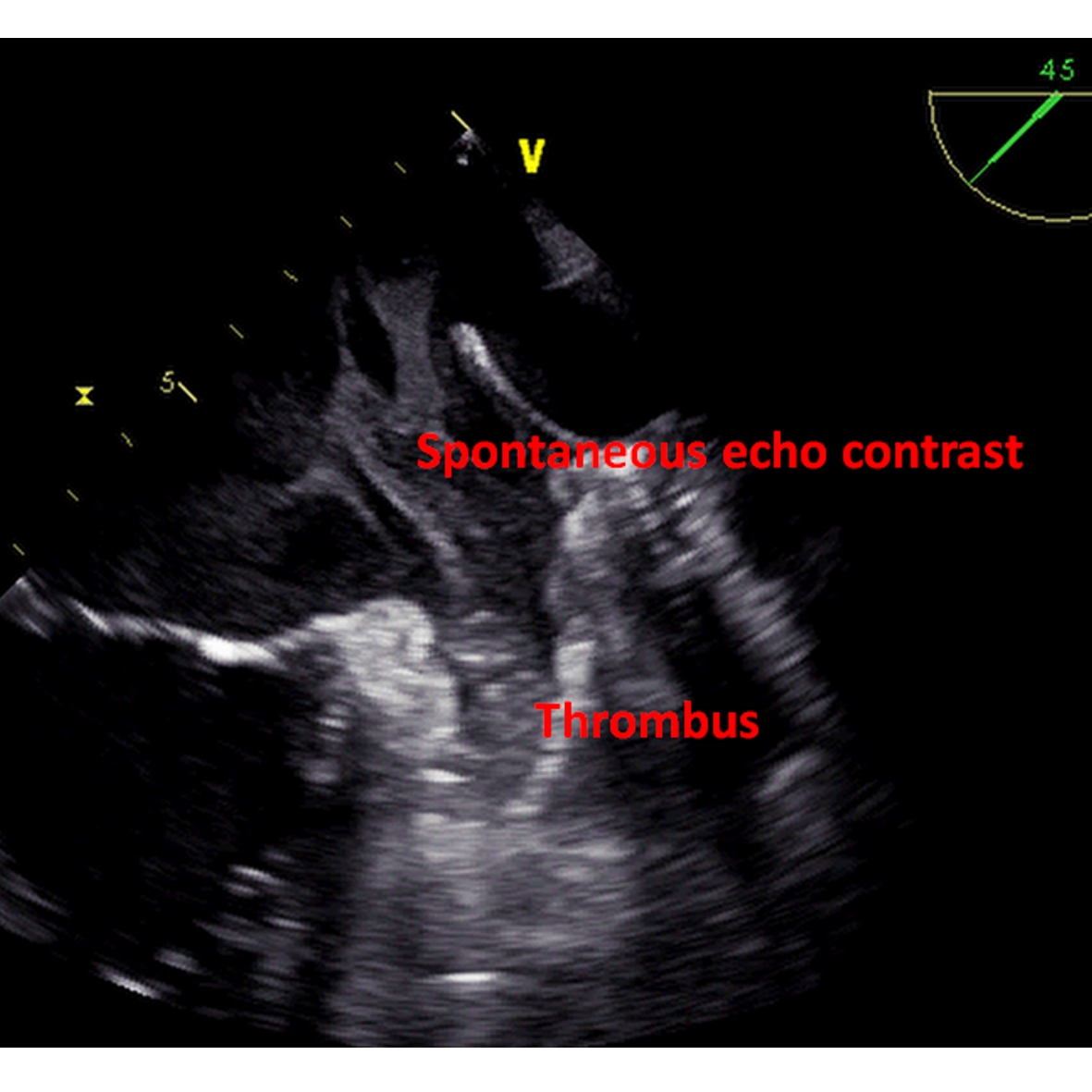

Supplement: Supplementary file 1 — Supporting information. [file CLC-46-397-s001.jpg]

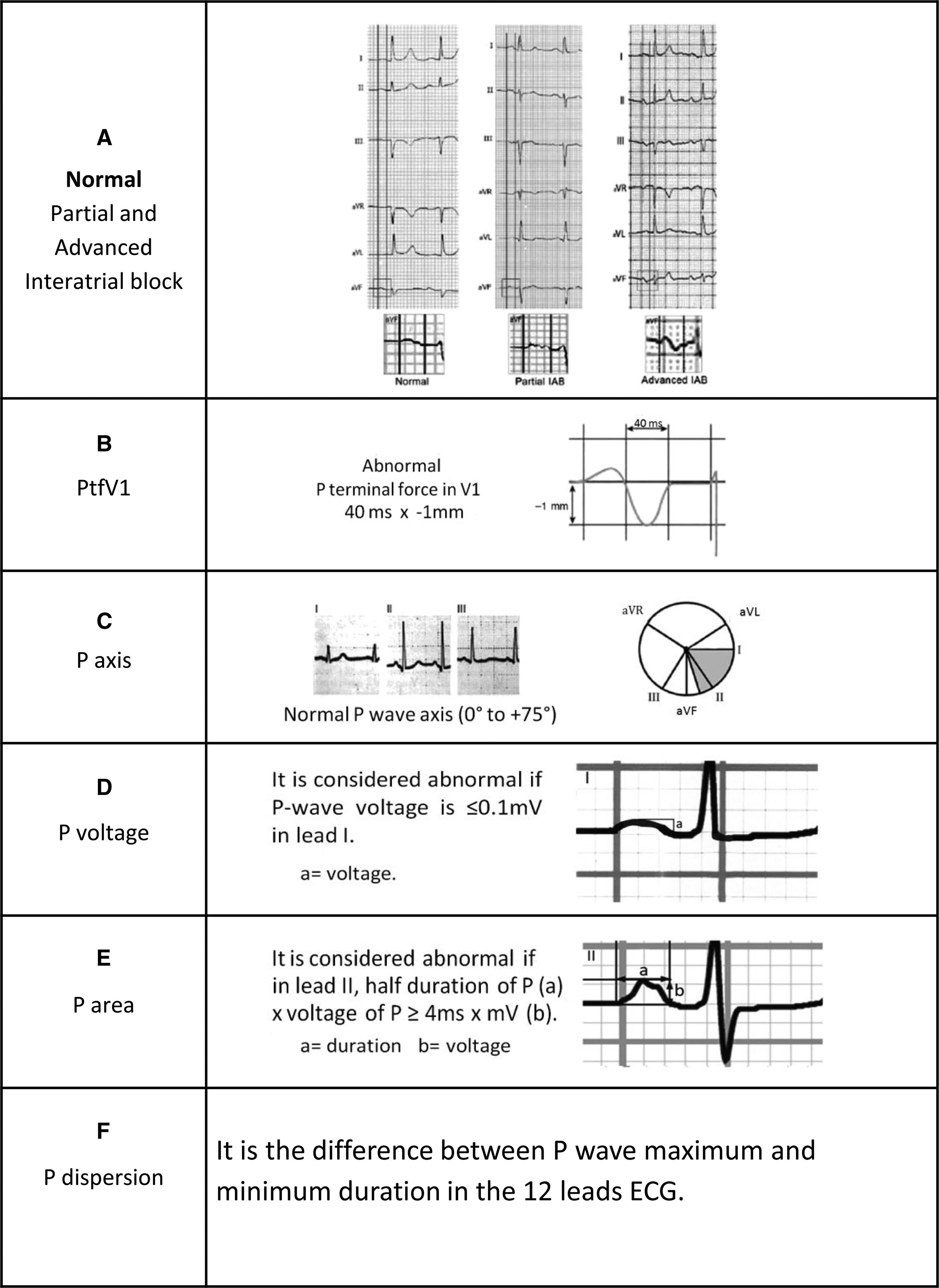

Supplement: Supplementary file 2 — Supporting information. [file CLC-46-397-s003.jpg]
