# Supplementary material for: P‐wave parameters and their association with thrombi and spontaneous echo contrast in the left atrial appendage
Source: Clin Cardiol. 2023 Feb 17;46(4):397–406. doi: 10.1002/clc.23980 (PMC10106666; doi:10.1002/clc.23980)
Supplement: Supplementary file 3 — Supporting information. [file CLC-46-397-s002.docx]

**Supplements: Subgroup analysis (patients with oral anticoagulation)**

**Supplements Table 1:** Characteristics of the subgroup (n = 91)

|  | Thrombus / SEC group  (n = 13) | Control group  (n = 78) | p value |
| --- | --- | --- | --- |
| Age (years) | 72 ± 9 | 70 ± 8 | 0.429 |
| CHA_2_DS_2_-VASc-Score, pts  Congestive heart failure, n (%) | 4.2 ± 1.6  6 (46) | 3.9 ± 1.1  18 (23) | 0.530  0.121 |
| Hypertension, n (%)  Diabetes mellitus, n (%)  Stroke / TIA, n (%)  Vascular disease, n (%) | 13 (100)  3 (23)  4 (31)  8 (62) | 72 (92)  13 (17)  14 (18)  37 (47) | 0.943  0.665  0.356  0.503 |
| Women (♀), n (%) | 6 (46) | 48 (62) | 0.296 |
| COPD, n (%) | 1 (8) | 10 (13) | 0.599 |
| Echocardiography |  |  |  |
| Left ventricular ejection fraction (%) | 49 ± 15 | 56 ± 10 | 0.042 |
| Left atrial diameter (mm) | 42 ± 6 | 42 ± 5 | 0.684 |
| Mitral valve regurgitation, n (%) | 11 (85) | 63 (81) | 0.946 |
| Labor |  |  |  |
| Creatinine (mg/dL)  Hemoglobin (g/dL) | 1.2 ± 0.4  12.3 ± 1.9 | 1.0 ± 0.2  13.4 ± 1.5 | 0.018  0.034 |

COPD*,* Chronic obstructive pulmonary disease; SEC, Spontaneous echo contrast

**Supplements Table 2:** ECG indices of the subgroup (n = 91)

|  | Thrombus / SEC group  (n = 13) | Control group  (n = 78) | p value |
| --- | --- | --- | --- |
| Heart rate (beats/min) | 65 ± 15 | 65 ± 11 | 0.979 |
| P-wave duration mean (ms) | 129 ± 12 | 113 ± 19 | 0.005 |
| P-wave axis (°) | 55 ± 22 | 53 ± 20 | 0.805 |
| P-wave dispersion (ms) | 45 ± 12 | 28 ± 14 | < 0.001 |
| P-wave amplitude in I < 0.1 mV, n (%) | 12 (92) | 51 (65) | 0.052 |
| P-wave area (mV*ms) | 7.8 ± 4.2 | 6.6 ± 2.8 | 0.216 |
| P-wave terminal force in V1 (µV*ms) | -8462 ± 5717 | -4814 ± 3067 | 0.001 |
| Partial interatrial block, n (%) | 8 (62) | 30 (38) | 0.118 |
| Advanced interatrial block, n (%) | 4 (31) | 12 (15) | 0.177 |
| PR interval (ms) | 189 ± 29 | 179 ± 35 | 0.339 |
| QRS duration (ms) | 108 ± 25 | 100 ± 19 | 0.162 |
| QT interval (ms) | 439 ± 49 | 422 ± 34 | 0.117 |
| QRS axis (°) | 9 ± 49 | 16 ± 37 | 0.544 |
| T-wave axis (°) | 45 ± 70 | 49 ± 35 | 0.739 |

SEC, Spontaneous echo contrast
